# Supplementary material for: The ancillary N-terminal region of the yeast AP-1 transcription factor Yap8 contributes to its DNA binding specificity
Source: Nucleic Acids Res. 2020 May 1;48(10):5426–41. doi: 10.1093/nar/gkaa316 (PMC7261193; doi:10.1093/nar/gkaa316)
Supplement: gkaa316_Supplemental_File [file gkaa316_supplemental_file.pdf]

SUPPLEMENTARY INFORMATION

The ancillary N-terminal region of the yeast AP-1 transcription factor Yap8 contributes to its DNA binding specificity

Ewa Maciaszczyk-Dziubinska[1], Anna Reymer[2], Nallani Vijay Kumar[2], Wojciech Białek[3], Katarzyna Mizio[1], Markus J. Tamás[2] and Robert Wysocki[1,*]

[1]Institute of Experimental Biology, University of Wroclaw, 50-328 Wroclaw, Poland

[2]Department of Chemistry and Molecular Biology, University of Gothenburg, Box 462, S-405 30 Gothenburg, Sweden

[3]Faculty of Biotechnology, University of Wroclaw, 50-383 Wroclaw, Poland

[*]To whom correspondence should be addressed. Tel: 48-71-375-4126; E-mail: robert.wysocki@uwr.edu.pl

Content:

Supplementary Methods, Tables S1-S4, Figures S1-S6

SUPPLEMENTARY METHODS

Details of molecular dynamics simulation protocols and analysis procedures

Molecular dynamics simulations (MD) were carried out with GROMACS MD software package, version 5.1 (1). Simulations were carried out using the combination of the latest AMBER all-atom nucleic acid Parmbsc1 (2) and ff14SB (3) force fields. Each protein-DNA complex was first neutralized with 34 K+ counterions and solvated in a 15Å solvent layer of SCP/E water molecules (4). Additional K+ and Cl- ions were added to reach overall KCl concentration of 150 mM. Each protein-DNA complex was initially energy minimized with 5000 steps of steepest descent, followed by a 500 ps simulation at constant volume, while raising the temperature to 300 K. Afterwards MD simulations were performed at constant pressure and temperature (1 atm., 300 K) using a weak-coupling thermostat (5) with a 0.2 ps coupling constant and an isotropic Parrinello-Rahman barostat (6) with a 2 ps coupling constant, and a 2 fs time step. Bonds involving hydrogen atoms were constrained with the LINCS algorithm (7) and the non-bonded pair list was updated every 20 fs with the group scheme (8). Electrostatic forces were evaluated using particle mesh Ewald algorithm (9) with a real-space cut-off of 10Å. The van der Waals forces were truncated at 10Å also and long-range corrections were added. Center of mass movement was removed every 0.2 ps to eliminate translational kinetic energy (10). Following the initial 100 ns of NPT MD, considered as equilibration, productive runs were recorded for another 0.5 μs for each setup.

Details of MD analysis procedure

MD trajectories were analyzed using CPPTRAJ program (11), with a particular focus on the protein-DNA interactions: including hydrogen bonds, salt bridges, and hydrophobic (apolar) interactions. The limit of a direct interaction was set up to be ≤ 3.5Å for a hydrogen bond between relevant heavy atoms, and the angle limit was set up to ≥ 135° at the intervening hydrogen atom. While for a salt bridge interaction the limit was set up to 3.5-6.0Å, bearing in mind possible interaction through a bridging water or a bridging counterion. A hydrophobic interaction was defined as a contact ≤ 6Å between the centers of mass of hydrophobic residues (Ala, Ile, Leu, Met, Phe, Trp) and DNA bases. The hydrogen bonds and salt bridges interactions were characterized by a fraction of the trajectory snapshots during which they were maintained, and by the average lifetimes (the lifetime calculations has been smoothed out by ignoring interruption of interactions shorter than 1 ps). Dynamic contacts maps were created by summing up the hydrogen bonds and the salt bridge interactions for each pair of Yap8-DNA interacting resides, which resulted in a contact strength value.

REFERENCES FOR SUPPLEMENTARY METHODS

1. James,M., Murtola,T., Schulz,R., Smith,J.C., Hess,B. and Lindahl,E. (2015) GROMACS : High performance molecular simulations through multi-level parallelism from laptops to supercomputers. *SoftwareX*, **2**, 19–25.
2. Ivani,I., Dans,P.D., Noy,A., Pérez,A., Faustino,I., Hospital,A., Walther,J., Andrio,P., Goñi,R., Balaceanu,A., *et al.* (2015) Parmbsc1: a refined force field for DNA simulations. *Nat. Methods*, **13**, 55–58.
3. Maier,J.A., Martinez,C., Kasavajhala,K., Wickstrom,L., Hauser,K.E. and

Simmerling,C. (2015) ff14SB: Improving the Accuracy of Protein Side Chain and Backbone Parameters from ff99SB. *J. Chem. Theory Comput.*, **11**, 3696–3713.

4. Mark,P. and Nilsson,L. (2001) Structure and Dynamics of the TIP3P, SPC, and SPC/E Water Models at 298 K. *J. Phys. Chem. A*, **105**, 9954–9960.
5. Berendsen,H.J.C., Postma,J.P.M., Gunsteren,W.F. Van, Dinola,A., Haak,J.R., Berendsen,H.J.C., Postma,J.P.M., Gunsteren,W.F. Van, Dinola,A. and Haak,J.R. (1984) Molecular dynamics with coupling to an external bath Molecular dynamics with coupling to an external bath. **81**, 3684–3690.
6. Parrinello,M. and Rahman,A. (1981) Polymorphic transitions in single crystals : A new molecular dynamics method. *J. Appl. Phys.*, **52**, 7182–7190.
7. Hess,B., Bekker,H., Berendsen,H.J.C. and Fraaije,J.G.E.M. (1997) LINCS : A Linear Constraint Solver for Molecular Simulations. **18**, 1463–1472.
8. Páll,S. and Hess,B. (2013) A flexible algorithm for calculating pair interactions on SIMD architectures. *Comput. Phys. Commun.*, **184**, 2641–2650.
9. Darden,T., York,D. and Pedersen,L. (1993) Particle mesh Ewald: An N· log(N) method for Ewald sums in large systems. *J. Chem. Phys.*, **98**, 10089–10092.
10. Harvey,S.C., Tan,R.K.-Z. and Cheatham,T.E. (1998) The flying ice cube: Velocity rescaling in molecular dynamics leads to violation of energy equipartition. *J. Comput. Chem.*, **19**, 726–740.
11. Roe,D.R. and Cheatham,T.E. (2013) PTRAJ and CPPTRAJ: Software for Processing and Analysis of Molecular Dynamics Trajectory Data. *J. Chem. Theory Comput.*, **9**, 3084–3095.

Supplementary Table S1

Table S1. Plasmids used in this study.

Plasmid name	Description	Reference
pYX122	CEN vector, *HIS3*, *TPI1* promoter	
pYX122-YAP8	*YAP8-HA* fusion cloned under *TPI1* promoter in pYX122	(1)
pYX122-YAP8-Δ5-13	*yap8-Δ5-13* mutation generated in pYX122-YAP8	This study
pYX122-YAP8-P4A	P4A mutation generated in pYX122-YAP8	This study
pYX122-YAP8-R5A	R5A mutation generated in pYX122-YAP8	This study
pYX122-YAP8-G6A	G6A mutation generated in pYX122-YAP8	This study
pYX122-YAP8-R7A	R7A mutation generated in pYX122-YAP8	This study
pYX122-YAP8-K8A	K8A mutation generated in pYX122-YAP8	This study
pYX122-YAP8-G9A	G9A mutation generated in pYX122-YAP8	This study
pYX122-YAP8-G10A	G10A mutation generated in pYX122-YAP8	This study
pYX122-YAP8-R11A	R11A mutation generated in pYX122-YAP8	This study
pYX122-YAP8-K12A	K12A mutation generated in pYX122-YAP8	This study
pYX122-YAP8-P13A	P13A mutation generated in pYX122-YAP8	This study
pYX122-YAP8-S14A	S14A mutation generated in pYX122-YAP8	This study
pYX122-YAP8-L15A	L15A mutation generated in pYX122-YAP8	This study
pYX122-YAP8-T16A	T16A mutation generated in pYX122-YAP8	This study
pYX122-YAP8-P17A	P17A mutation generated in pYX122-YAP8	This study
pYX122-YAP8-P18A	P18A mutation generated in pYX122-YAP8	This study
pYX122-YAP8-N20A	N20A mutation generated in pYX122-YAP8	This study
pYX122-YAP8-N20Q	N20Q mutation generated in pYX122-YAP8	This study
pYX122-YAP8-N20D	N20D mutation generated in pYX122-YAP8	This study
pYX122-YAP8-K21A	K21A mutation generated in pYX122-YAP8	This study
pYX122-YAP8-Q25A	Q25A mutation generated in pYX122-YAP8	This study
pYX122-YAP8-F33A	F33A mutation generated in pYX122-YAP8	This study
pYX122-YAP8-K35A	K35A mutation generated in pYX122-YAP8	This study
pYX122-YAP8-R36A	R36A mutation generated in pYX122-YAP8	This study
pYX122-YAP8-K37A	K37A mutation generated in pYX122-YAP8	This study
pYX122-YAP8-L38A	L38A mutation generated in pYX122-YAP8	This study
pYX122-YAP8-E39A	E39A mutation generated in pYX122-YAP8	This study
pYX122-YAP8-R40A	R40A mutation generated in pYX122-YAP8	This study
pYX122-YAP8-4aa	Quadruple (A23T L26N S29A N31R) mutation generated in pYX122-YAP8	This study
pYX122-YAP8-7aa	Septuple (A23T L26N S29A N31R K35E L37E E39R) mutation generated in pYX122-YAP8	This study
pYX122-YAP8-8aa	Octuple (N20Q A23T L26N S29A N31R K35E L37E E39R) mutation generated in pYX122-YAP8	This study
pYX122-YAP1	*YAP1-HA* fusion cloned under *TPI1* promoter in pYX122	This study
pGEX4T-1-GST-YAP8	*GST-YAP8* fusion for expression in *E. coli*	(2)
pGST-YAP8-N20A	N20A mutation generated in pGEX4T-1-GST-YAP8	This study
pGST-YAP8-N20Q	N20Q mutation generated in pGEX4T-1-GST-YAP8	This study
pGST-YAP8-Q25A	Q25A mutation generated in pGEX4T-1-GST-YAP8	This study
pGST-YAP8-4aa	Quadruple (A23T L26N S29A N31R) mutation generated in pGEX4T-1-GST-YAP8	This study
pGST-YAP8-7aa	Septuple (A23T L26N S29A N31R K35E L37E E39R)	This study

	mutation generated in pGEX4T-1-GST-YAP8	
pGST-YAP8-8aa	Octuple (N20Q A23T L26N S29A N31R K35E L37E E39R) mutation generated in pGEX4T-1-GST-YAP8	This study
pGST-YAP8-R7A	R7A mutation generated in pGEX4T-1-GST-YAP8	This study
pGST-YAP8-R7K	R7K mutation generated in pGEX4T-1-GST-YAP8	This study
pGST-YAP8-G10A	G10A mutation generated in pGEX4T-1-GST-YAP8	This study
pGST-YAP8-R11A	R11A mutation generated in pGEX4T-1-GST-YAP8	This study
pGST-YAP8-R11K	R11K mutation generated in pGEX4T-1-GST-YAP8	This study
pGST-YAP8-R7K R11K	R7K and R11K mutations generated in pGEX4T-1-GST-YAP8	This study
pSEYC102	CEN vector, *URA3, lacZ* reporter gene	(3)
pEM19	*ACR3-lacZ* fusion in pSEYC102	(4)
pDP1	*MUT3-ACR3-lacZ* fusion in pSEYC102	(2)

SUPPLEMENTARY REFERENCES FOR TABLE S1

1. Di,Y. and Tamás,M.J. (2007) Regulation of the arsenic-responsive transcription factor Yap8p involves the ubiquitin-proteasome pathway. *J. Cell Sci.*, **120,** 256–264.
2. Ilina,Y., Sloma,E., Maciaszczyk-Dziubinska,E., Novotny,M., Thorsen,M., Wysocki,R. and Tamás,M. J. (2008) Characterization of the DNA-binding motif of the arsenic-responsive transcription factor Yap8p. *Biochem. J.,* **415,** 467–475.
3. Emr,S.D., Vassarotti,A., Garrett,J., Geller,B.L., Takeda,M. and Douglas,M.G. (1986) The amino terminus of the yeast F_1-ATPase beta-subunit precursor functions as a mitochondrial import signal. *J. Cell Biol*., **102,** 523–533.
4. Wysocki,R., Fortier,P.K., Maciaszczyk,E., Thorsen,M., Leduc,A., Odhagen,A., Owsianik,G., Ulaszewski,S., Ramotar,D. and Tamás,M.J. (2004) Transcriptional activation of metalloid tolerance genes in *Saccharomyces cerevisiae* requires the AP-1-like proteins Yap1p and Yap8p. *Mol. Biol. Cell*, **15,** 2049–2060.

Supplementary Table S2

Table S2. Oligonucleotides used in this work.

Name	Sequence 5' to 3'
**Mutagenesis:**	
P4A-fw	CATGGGTATGGCAAAAGCGCGTGGAAGAAAAGG
P4A-rv	CCTTTTCTTCCACGCGCTTTTGCCATACCCATG
R5A-fw	GGTATGGCAAAACCGGCTGGAAGAAAAGGCGGC
R5A-rv	GCCGCCTTTTCTTCCAGCCGGTTTTGCCATACC
G6A-fw	GTATGGCAAAACCGCGTGCAAGAAAAGGCGGCAGG
G6A-rv	CCTGCCGCCTTTTCTTGCACGCGGTTTTGCCATAC
R7A-fw	GGCAAAACCGCGTGGAGCAAAAGGCGGCAGGAAG
R7A-rv	CTTCCTGCCGCCTTTTGCTCCACGCGGTTTTGCC
R7K-fw	CATGGCAAAACCGCGTGGAAAAAAAGGCGGCAGGAAGCC
R7K-rv	GGCTTCCTGCCGCCTTTTTTTCCACGCGGTTTTGCCATG
K8A-fw	CAAAACCGCGTGGAAGAGCAGGCGGCAGGAAGCCTC
K8A-rv	GAGGCTTCCTGCCGCCTGCTCTTCCACGCGGTTTTG
G9A-fw	CGCGTGGAAGAAAAGCCGGCAGGAAGCCTTCAC
G9A-rv	GTGAAGGCTTCCTGCCGGCTTTTCTTCCACGCG
G10A-fw	CGTGGAAGAAAAGGCGCCAGGAAGCCTTCACTTAC
G10A-rv	GTAAGTGAAGGCTTCCTGGCGCCTTTTCTTCCACG
R11A-fw	GGAAGAAAAGGCGGCGCGAAGCCTTCACTTACTC
R11A-rv	GAGTAAGTGAAGGCTTCGCGCCGCCTTTTCTTCC
R11K-fw	GTGGAAGAAAAGGCGGCAAGAAGCCTTCACTTACTCC
R11K-rv	GGAGTAAGTGAAGGCTTCTTGCCGCCTTTTCTTCCAC
R7K R11K-fw	GCAAAACCGCGTGGAAAAAAAGGCGGCAAGAAGCCTTCACTTACTCC
R7K R11K-rv	GGAGTAAGTGAAGGCTTCTTGCCGCCTTTTTTTCCACGCGGTTTTGC
K12A-fw	GAAAAGGCGGCAGGGCGCCTTCACTTACTCCAC
K12A-rv	GTGGAGTAAGTGAAGGCGCCCTGCCGCCTTTTC
P13A-fw	GAAAAGGCGGCAGGAAGGCTTCACTTACTCCACC
P13A-rv	GGTGGAGTAAGTGAAGCCTTCCTGCCGCCTTTTC
S14A-fw	GGCGGCAGGAAGCCTGCACTTACTCCACCTAAA
S14A-rv	TTTAGGTGGAGTAAGTGCAGGCTTCCTGCCGCC
L15A-fw	GGCGGCAGGAAGCCTTCAGCTACTCCACCTAAAAA
L15A-rv	TTTTTAGGTGGAGTAGCTGAAGGCTTCCTGCCGCC
T16A-fw	CAGGAAGCCTTCACTTGCTCCACCTAAAAATAAGAG
T16A-rv	CTCTTATTTTTAGGTGGAGCAAGTGAAGGCTTCCTG
P17A-fw	GGAAGCCTTCACTTACTGCACCTAAAAATAAGAGAGC
P17A-rv	GCTCTCTTATTTTTAGGTGCAGTAAGTGAAGGCTTCC
P18A-fw	GCCTTCACTTACTCCAGCTAAAAATAAGAGAGCTGCG
P18A-rv	CGCAGCTCTCTTATTTTTAGCTGGAGTAAGTGAAGGC
K19A-fw	CCTTCACTTACTCCACCTGCAAATAAGAGAGCTGCGCAAC
K19A-rv	GTTGCGCAGTCTCTTATTTGCAGGTGGAGTAAGTGAAGG
N20A-fw	CACTTACTCCACCTAAACAAAAGAGAGCTGCGCAACTTAGAG
N20A-rv	CTCTAAGTTGCGCAGCTCTCTTTTGTTTAGGTGGAGTAAGTG
N20D-fw	CACTTACTCCACCTAAAGACAAGAGAGCTGCGCAACTTAGAG
N20D-rv	CTCTAAGTTGCGCAGCTCTCTTGTCTTTAGGTGGAGTAAGTG
N20Q-fw	CACTTACTCCACCTAAACAAAAGAGAACTGCGCAAAATAGAGC
N20Q-rv	GCTCTATTTTGCGCAGTTCTCTTTTGTTTAGGTGGAGTAA GTG
K21A-fw	CTTACTCCACCTAAAAATGCGAGAGCTGCGCAACTTAGAG
K21A-rv	CTCTAAGTTGCGCAGCTCTCGCATTTTTAGGTGGAGTAAG
A23T-fw	CCACCTAAAAATAAGAGAACTGCGCAACTTAGAGCATC
A23T-rv	GATGCTCTAAGTTGCGCAGTTCTCTTATTTTTAGGTGG

Q25A-fw	CTAAAAATAAGAGAGCTGCGGCACTTAGAGCATCCC
Q25A-rv	GGGATGCTCTAAGTGCCGCAGCTCTCTTATTTTTAG
N31R-fw	CTGCGCAACTTAGAGCATCCCAAAGAGCATTTAGAAAACG
N31R-rv	CGTTTTCTAAATGCTCTTTGGGATGCTCTAAGTTGCGCAG
F33A-fw	CTTAGAGCATCCCAAAACGCAGCTAGAAAACGAAAGTTGG
F33-rv	CCAACTTTCGTTTTCTAGCTGCGTTTTGGGATGCTCTAAG
A23TL26NS29A-fw	CCACCTAAAAATAAGAGAACTGCGCAAAATAGAGCAGC
A23TL26NS29A-rv	GCTGCTCTATTTTGCGCAGTTCTCTTATTTTTAGGTGG
A23TL26NS29AN31R-fw	GAACTGCGCAAAATAGAGCAGCTCAAAGAGCATTTAGAAAACG
A23TL26NS29AN31R-rv	CGTTTTCTAAATGCTCTTTGAGCTGCTCTATTTTGCGCAGTTC
K35EL37EE39R-fw	CAAAGAGCATTTAGAGAACGAAAGGAGCGAAGATTAGAAGAACTAG
K35EL37EE39R-rv	CTAGTTCTTCTAATCTTCGCTCCTTTCGTTCTCTAAATGCTCTTTG
K35A-fw	GCATCCCAAAACGCATTTAGAGCACGAAAGTTGGAAAGATTAG
K35A-rv	CTAATCTTTCCAACTTTCGTGCTCTAAATGCGTTTTGGGATGC
R36A-fw	CCCAAAACGCATTTAGAAAAGCAAAGTTGGAAAGATTAGAAG
R36A-rv	CTTCTAATCTTTCCAACTTTGCTTTTCTAAATGCGTTTTGGG
K37A-fw	CCCAAAACGCATTTAGAAAACGAGCGTTGGAAAGATTAGAAG
K37A-rv	CTTCTAATCTTTCCAACGCTCGTTTTCTAAATGCGTTTTGGG
L38A-fw	CGCATTTAGAAAACGAAAGGCGGAAAGATTAGAAGAACTAGAG
L38A-rv	CTCTAGTTCTTCTAATCTTTCCGCCTTTCGTTTTCTAAATGCG
E39A-fw	GCATTTAGAAAACGAAAGTTGGCAAGATTAGAAGAACTAGAGAAG
E39A-rv	CTTCTCTAGTTCTTCTAATCTTGCCAACTTTCGTTTTCTAAATGC
R40A-fw	GAAAACGAAAGTTGGAAGCATTAGAAGAACTAGAGAAGAAG
R40A-rv	CTTCTTCTCTAGTTCTTCTAATGCTTCCAACTTTCGTTTTC
**qRT-PCR:**	
ACR3-fw	CGGCATACCACTGGGAATT
ACR3-rv	GCACCAATGGGACAAAGCA
PRACR3-fw	TTACGCTTGCTGGATTGTCA
PRACR3-rv	CGTTGCCGCTAAAGTTGATT
IPP1-fw	CTTTATTGGATGAAGGTGA
IPP1-rv	TTAATTGTTTCCAGGAGTC
**EMSA (biotin-labelled):**	
ACR3short-fw	TCTTAATTATCTTTTTGTTTGATTAATAATCAACTTTAGCGGCAACGCTCC
ACR3short-rv	GGAGCGTTGCCGCTAAAGTTGATTATTAATCAAACAAAAAGATAATTAAGA
MUT3short-fw	TCTTAATTATCTTTTTGTTTGATTACTAATCAACTTTAGCGGCAACGCTCC
MUT3short-rv	GGAGCGTTGCCGCTAAAGTTGATTAGTAATCAAACAAAAAGATAATTAAGA
ACR3-M1-fw	TCTTAATTATCTTTGCGGCTGATTAATAATCAACTTTAGCGGCAACGCTCC
ACR3-M1-rv	GGAGCGTTGCCGCTCCGGTTGATTATTAATCAAACAAAAAGATAATTAAGA
ACR3-M2-fw	TCTTAATTATCTTTTTGTTTGATTAATAATCAACCGGAGCGGCAACGCTCC
ACR3-M2-rv	GGAGCGTTGCCGCTCCGGTTGATTATTAATCAAACAAAAAGATAATTAAGA
ACR3-M3-fw	TCTTAATTATCTTTGCGGCTGATTAATAATCAACCGGAGCGGCAACGCTCC
ACR3-M3-rv	GGAGCGTTGCCGCTCCGGTTGATTATTAATCAGCCGCAAAGATAATTAAGA
TRX2short-fw	ATTGTTTATACTCTTAGTAAAGGATGCTCCCTACAAGGTGGCTCTTTTCTTACTAAGCGCGTTCAGTTTC

TRX2short-rv	GAAACTGAACGCGCTTAGTAAGAAAAGAGCCACCTTGTAGGGAGCATCCTTTACTAAGAGTATAAACAAT
GSH1short-fw	TTCTGCCCAACGACGGCTGCCATTAGTCAGCATGGCGCGCACGTGACTACA
GSH1short-rv	TGTAGTCACGTGCGCGCCATGCTGACTAATGGCAGCCGTCGTTGGGCAGAA
**Fluorescence anisotropy (FAM-labelled):**	
WT-ACR3	CTTTTTGTTTGATTAATAATCAACTTTAGCG
ACR3-M3	CTTTGCGGCTGATTAATAATCAACCGGAGCG

Supplementary Table S3

Table S3. Interactions between Yap8 wild-type protein dimer and DNA characterized by the percentage presence during 0.5 μs MD simulation and the average lifetime (<LT>)(ps). Interactions in orange represent the salt bridges, in black – the hydrogen bonds between the DNA backbone and the protein, and in blue – the hydrogen bonds between the DNA bases and the protein. The table is limited to the interactions that occur at least 5% of the time of the MD simulation.

Contact name	%	<LT> (ps)	Contact name	%	<LT> (ps)
**Yap8 wild-type monomer 1**					
T6$_{w}$ (OP2)-Arg11 (N)	99	2157	T2$_{w}$ (O2)-Arg7 (NH1)	26	15
T10$_{w}$ (OP1)-Arg36 (NH1)	99	868	A3$_{c}$ (OP2)-Arg11 (NH2)	25	196
A9$_{w}$ (OP1)-Ser29 (OG)	94	564	T7$_{w}$ (OP2)-Arg11 (NE)	19	52
T6$_{w}$ (OP2)-Lys12 (N)	93	612	A14$_{c}$ (OP1)-Arg34 (NH2)	16	22
T3$_{w}$ (O2)-Arg7 (NH2)	91	1006	T12$_{c}$ (O5')-Arg27 (NH1)	15	27
T5$_{w}$ (O3')-Gly10 (N)	87	501	G8$_{w}$ (O5')-Asn25 (NE2)	14	13
A1$_{c}$ (OP2)-Lys8 (N)	81	82	G8$_{w}$ (OP2)-Asn25 (NE2)	14	12
T5$_{w}$ (OP2)-Arg7 (N)	72	66	T7$_{w}$ (OP2)-Arg11 (NH1)	14	26
A11$_{c}$ (N6)-Gln30 (OE1)	72	53	T12$_{c}$ (OP1)-Arg27 (NE)	14	30
A2$_{c}$ (O3')-Lys8 (N)	69	34	T7$_{w}$ (OP1)-Arg22 (NH2)	14	22
G8$_{w}$ (O6)-Arg22 (NH1)	51	71	A1$_{c}$ (OP1)-Lys8 (NZ)	13	13
A3$_{c}$ (OP2)-Arg11 (NH1)	50	38	T13$_{c}$ (OP1)-Arg34 (NH2)	11	40
G8$_{w}$ (N7)-Arg22 (NH2)	49	94	T5$_{w}$ (OP2)-Lys12 (NZ)	10	23
T7$_{w}$ (OP2)-Arg11 (NH2)	44	79	G8$_{w}$ (N7)-Arg22 (NH1)	9	13
G8$_{w}$ (OP1)-Asn25 (NE2)	43	22	T13$_{c}$ (OP1)-Arg34 (NH1)	9	27
T3$_{w}$ (O2)-Arg7 (NH1)	40	17	T7$_{w}$ (OP2)-Arg22 (NH1)	8	13
T12$_{c}$ (OP1)-Arg27 (NH1)	38	30	T3$_{w}$ (O4')-Arg7 (NH1)	8	8
T7$_{w}$ (OP1)-Arg22 (NH1)	36	92	G8$_{w}$ (O6)-Arg22 (NH2)	7	16
A3$_{c}$ (OP2)-Arg11 (NE)	33	166	T12$_{c}$ (O5')-Arg27 (NH2)	7	14
T10$_{w}$ (O4)-Gln30 (NE2)	33	96	T6$_{w}$ (O3')-Arg11 (NH2)	7	20
A14$_{c}$ (OP1)-Arg34 (NH1)	31	48	T7$_{w}$ (OP1)-Arg11 (NE)	7	20
A11$_{c}$ (N6)-Gln30 (NE2)	29	20	T7$_{w}$ (OP1)-Arg11 (NH2)	6	48
T6$_{w}$ (OP1)-Lys12 (NZ)	28	51	A11$_{c}$ (N7)-Gln30 (NE2)	6	27
T12$_{c}$ (OP1)-Arg27 (NH2)	28	81	A11$_{c}$ (OP1)-Arg27 (NH1)	6	53
C4$_{c}$ (O3')-Arg11 (NH1)	28	24	A14$_{c}$ (O5')-Arg34 (NH2)	5	11
**Yap8 wild-type monomer 2**					
T15$_{c}$ (O4)-Gln30 (NE2)	100	922	A13$_{w}$ (N6)-Arg34 (NH2)	14	18
G18$_{c}$ (O6)-Arg22 (NH1)	91	154	A17$_{c}$ (OP1)-Ser29 (OG)	14	33
T19$_{c}$ (OP1)-Lys21 (NZ)	82	90	T19$_{c}$ (OP2)-Arg11 (NE)	12	41
T14$_{w}$ (OP1)-Arg27 (NE)	80	694	A13$_{w}$ (OP2)-Arg27 (NE)	11	30
T14$_{w}$ (OP1)-Arg27 (NH1)	78	172	T24$_{w}$ (OP1)-Lys8 (NZ)	11	13
T24$_{w}$ (OP2)-Lys8 (N)	60	64	A12$_{w}$ (O5')-Arg34 (NH2)	11	13
G18$_{c}$ (N7)-Arg22 (NH2)	56	29	T24$_{w}$ (OP1)-Lys8 (N)	11	38

Contact name	%	<LT> (ps)	Contact name	%	<LT> (ps)
G18$_c$ (O6)-Arg22 (NH2)	54	27	T19$_c$ (OP2)-Arg11 (N)	10	263
A17$_c$ (OP1)-Arg36 (NH2)	54	58	A25$_w$ (OP2)-Arg7 (NH1)	10	26
T16$_c$ (OP1)-Arg36 (NH1)	53	56	T23$_w$ (OP1)-Lys8 (NZ)	9	20
T22$_w$ (O2)-Arg7 (NH1)	50	202	T23$_w$ (OP2)-Arg11 (NE)	9	42
A12$_w$ (OP1)-Arg34 (NH1)	50	135	T23$_w$ (OP2)-Arg11 (NH1)	9	22
T19$_c$ (OP2)-Ser14 (OG)	43	169	T23$_w$ (OP2)-Lys8 (NZ)	9	16
T23$_w$ (O3')-Lys8 (N)	40	28	A25$_w$ (OP1)-Arg7 (NE)	8	26
T14$_w$ (O4)-Gln30 (NE2)	37	21	A13$_w$ (O5')-Arg27 (NE)	8	15
T19$_c$ (OP1)-Asn25 (NE2)	35	75	A22$_c$ (N3)-Arg7 (NH2)	7	53
A13$_w$ (N7)-Arg34 (NH2)	34	69	A23$_c$ (N3)-Arg7 (NH2)	7	601
T14$_w$ (OP2)-Arg27 (NH1)	33	12	A13$_w$ (OP1)-Arg27 (NE)	7	15
T22$_w$ (O2)-Arg7 (NE)	32	48	T24$_w$ (OP1)-Gly9 (N)	7	50
A13$_w$ (OP1)-Asn31 (ND2)	30	67	A15$_w$ (OP1)-Lys19 (NZ)	6	25
A17$_c$ (OP1)-Arg36 (NH1)	25	39	T23$_w$ (OP2)-Arg11 (NH2)	6	28
T19$_c$ (O5')-Asn25 (NE2)	25	23	T20$_c$ (O3')-Ser14 (OG)	6	12
A12$_w$ (OP1)-Arg34 (NH2)	24	36	T23$_w$ (OP1)-Arg11 (NH1)	6	25
G18$_c$ (OP1)-Asn25 (NE2)	23	119	A23$_c$ (O4')-Arg7 (NH1)	6	41
A12$_w$ (OP1)-Arg34 (NE)	21	30	G18$_c$ (N7)-Arg22 (NH1)	6	25
T19$_c$ (OP2)-Arg11 (NH1)	21	52	T19$_c$ (O5')-Arg11 (NH1)	6	20
A13$_w$ (OP2)-Arg27 (NH1)	17	200	A25$_w$ (OP1)-Arg7 (NH1)	5	21
A13$_w$ (OP1)-Arg34 (NH1)	15	32	T16$_c$ (OP1)-Arg36 (NE)	5	11
T16$_c$ (OP1)-Arg36 (NH2)	15	155			

Supplementary Table S4

Table S4. Interactions between Yap8 Asn20Ala mutant protein dimer and DNA characterized by the percentage presence during 0.5 μs MD simulation and the average lifetime (<LT>)(ps). Interactions in orange represent the salt bridges, in black – the hydrogen bonds between the DNA backbone and the protein, and in blue – the hydrogen bonds between the DNA bases and the protein. The table is limited to the interactions that occur at least 5% of the time of the MD simulation.

Contact name	%	<LT> (ps)	Contact name	%	<LT> (ps)
**Yap8 Asn20Ala mutant monomer 1**					
A9$_w$ (OP1)-Ser29 (OG)	100	2080	A11$_c$ (N6)-Gln30 (NE2)	20	12
T10$_w$ (OP1)-Arg36 (NH1)	99	737	A11$_c$ (N6)-Gln30 (NE2)	20	12
T10$_w$ (O4)-Gln30 (NE2)	98	1018	T12$_c$ (O5')-Arg27 (NH2)	20	16
T3$_w$ (O2)-Arg7 (NH2)	89	194	T2$_w$ (O2)-Arg7 (NH1)	16	14
T7$_w$ (OP1)-Arg22 (NH1)	83	144	T5$_w$ (O3')-Lys12 (NZ)	14	15
T12$_c$ (OP1)-Arg27 (NH2)	75	80	T6$_w$ (OP1)-Lys12 (NZ)	14	83
T12$_c$ (OP1)-Arg27 (NH1)	68	53	A2$_c$ (O3')-Gly9 (N)	14	15
T7$_w$ (OP1)-Arg22 (NE)	66	36	A2$_c$ (O3')-Lys8 (N)	13	13
A1$_c$ (OP2)-Lys8 (N)	65	80	A2$_c$ (OP2)-Arg11 (N)	12	115
T3$_w$ (O2)-Arg7 (NH1)	59	25	T5$_w$ (OP2)-Lys12 (NZ)	11	41
T5$_w$ (OP2)-Arg7 (N)	54	68	A2$_c$ (OP2)-Lys12 (NZ)	10	31
A11$_c$ (N6)-Gln30 (OE1)	48	22	A1$_c$ (OP1)-Lys8 (NZ)	9	14
A14$_c$ (OP1)-Arg34 (NH1)	45	35	A3$_c$ (OP2)-Lys12 (NZ)	8	87
G8$_w$ (O5')-Gln25 (NE2)	45	33	T13$_c$ (OP1)-Arg34 (NH2)	7	49
T7$_w$ (O5')-Arg22 (NE)	38	16	C4$_c$ (O2)-Lys12 (NZ)	7	71
A14$_c$ (OP1)-Arg34 (NH2)	31	45	T6$_w$ (OP1)-Arg22 (NH1)	7	21
A1$_c$ (OP2)-Gly9 (N)	28	45	T3$_w$ (O4')-Arg7 (NH1)	6	8
T6$_w$ (OP2)-Lys12 (NZ)	28	34	A11$_c$ (OP1)-Arg27 (NH1)	6	55
G8$_w$ (OP1)-Gln25 (NE2)	27	25	T6$_w$ (OP2)-Arg22 (NH1)	5	22
T12$_c$ (O5')-Arg27 (NH1)	24	23	A1$_c$ (O3')-Arg7 (NE)	5	25
**Yap8 Asn20Ala mutant monomer 2**					
T15$_c$ (O4)-Gln30 (NE2)	100	1187	A16$_w$ (N7)-Arg22 (NH1)	13	22
A12$_w$ (OP1)-Arg34 (NH2)	91	317	T22$_w$ (O2)-Arg7 (NH1)	13	64
A17$_c$ (OP1)-Ser29 (OG)	89	740	T20$_c$ (OP2)-Arg7 (NH1)	12	40
T16$_c$ (OP1)-Arg36 (NH1)	87	1810	T19$_c$ (OP2)-Lys21 (NZ)	12	11
T19$_c$ (OP1)-Gln25 (NE2)	85	46	T20$_c$ (OP2)-Lys12 (NZ)	12	27
T19$_c$ (O5')-Gln25 (NE2)	80	37	A13$_w$ (O5')-Arg27 (NE)	12	14
A12$_w$ (OP1)-Arg34 (NH1)	80	83	T20$_c$ (OP2)-Arg11 (NH2)	10	58
T14$_w$ (OP1)-Arg27 (NE)	66	550	A17$_c$ (N7)-Ser29 (OG)	10	104
G18$_c$ (O6)-Arg22 (NH1)	63	52	T17$_w$ (O4)-Arg22 (NH1)	8	12
T14$_w$ (OP1)-Arg27 (NH1)	63	123	T24$_w$ (OP1)-Lys8 (NZ)	8	23
T24$_w$ (OP2)-Lys8 (N)	60	172	T23$_w$ (OP1)-Lys8 (NZ)	8	14
T19$_c$ (OP1)-Lys21 (NZ)	57	32	T17$_w$ (O4)-Arg22 (NH2)	7	137

**Contact name**	**%**	**<LT> (ps)**	**Contact name**	**%**	**<LT> (ps)**
T14$_w$ (O4)-Gln30 (NE2)	56	22	T20$_c$ (OP2)-Arg7 (NH2)	7	46
G18$_c$ (O6)-Arg22 (NE)	55	85	T20$_c$ (OP2)-Arg11 (NH1)	7	42
A13$_w$ (OP1)-Asn31 (ND2)	52	75	T16$_c$ (OP1)-Arg36 (NH2)	7	89
T24$_w$ (OP2)-Arg7 (NH2)	29	347	T23$_w$ (O3')-Lys8 (N)	7	27
A13$_w$ (OP2)-Arg27 (NH1)	29	159	A17$_c$ (O5')-Ser29 (OG)	7	7
A12$_w$ (O5')-Arg34 (NH1)	25	11	A25$_w$ (OP2)-Arg7 (NE)	6	47
T23$_w$ (OP2)-Lys8 (NZ)	24	16	T24$_w$ (OP2)-Arg7 (NE)	6	57
A25$_w$ (N3)-Arg7 (NH2)	23	82	G18$_c$ (O3')-Ser29 (OG)	6	6
T14$_w$ (OP2)-Arg27 (NH1)	23	11	A13$_w$ (N7)-Arg34 (NH1)	6	33
G18$_c$ (O6)-Arg22 (NH2)	23	104	A25$_w$ (N3)-Arg7 (NE)	5	50
A13$_w$ (OP1)-Arg27 (NE)	19	23	T24$_w$ (OP1)-Lys8 (N)	5	22
A13$_w$ (OP2)-Arg27 (NE)	16	26			

Supplementary Figure S1

```
                                     RxxxNxxAQxxFR
Sc_Yap1      51 KKGSKTSKKQDLDPETKQKRTAQNRAAQRAFRERKERK  88  (NP_013707)
Sk_Yap1      51 KKGTKISKKQDLDPETKQKRTAQNRAAQRAFRERKERK  88  (EJT43841)
Cg_Yap1      12 SSASRKKRYQELDPETRMKRVAQNRAAQKAFRERKERK  49  (XP_446996)
Ca_Cap1       7 STATTKVGRKPIDTEPKSKRTAQNRAAQRAYRERKERK  64  (EEQ44283)
Kl_Yap1      38 KRRERKPGRKPLETEAKDKRTAQNRAAQRAFRERRERK  75  (CAH02665)
Sc_Yap2      30 KRKVGRPGRKRIDSEAKSRRTAQNRAAQRAFRDRKEAK  67  (NP_010711)
Cg_Yap2       6 KEGKKKAGRKIIDTEAKNKRTAQNRAAQRAFRERKEAK  43  (XP_446103)
An_NapA     141 DKTSKKPGRKPLTSEPTSKRKAQNRAAQRAFRERKEKH 178  (XP_680782)
Cn_Bap1     144 RKSGGGEGDGKRELSKSERRKEQNRAAQKAFRERREAK 180  (XP_012046219)

Sc_Yap4     224 YNNDGQLIGKTGKPLRNTKRAAQNRSAQKAFRQRREKY 261  (NP_014671)
Sc_Yap6     208 PDKDTQLISSSGKTLRNTRRAAQNRTAQKAFRQRKEKY 245  (NP_010545)
Sk_Yap4/6   204 VERNIQQVGGSGKALRNTRRAAQNRTAQKAFRQRKEKY 241  (EJT44162)
Kl_Yap4/6   430 LNTEGELIGKSGKALRNTKRAAQNRNAQRAFRQRREKY 467  (CAG99797)
Cg_Yap4/6   145 TLLTSSAQLQGQKTKKNTKRAMQNRKAQKAFRQRKEQY 181  (XP_449728)
Ca_Cap4      75 KVDPELPKTHGHRIVSTTKRAEQNRNAQRAFRIRKANQ 112  (AOW27392)
An_ZipC      90 TVEDETPKTYGKRPLSTSKRAAQNRAAQRAFRQRKESY 127  (P0C5H8)
Cn_Yap4      17 RGDSAQPEDIHGRGSSSSARKAQNRIAQREFRLRKQQY  54  (XP_012052390)

Sc_Yap3     131 SRSPSVAHNENVPDDSKAKKKAQNRAAQKAFRERKEAR 168  (NP_011854)
Sk_Yap3     132 SVALSLFDEENIPGDFKAKKKAQNRAAQRAFRERKEAR 169  (EJT43941)
Cg_Yap3     179 SGKDKFNEEGLTEDEIKARKKAQNRAAQKAFRERKEAR 216  (XP_448338)
Cg_Yap3b    151 STFSHHRENSNPGDNIDDKKKAQNRAAQRAFRERKEAK 188  (XP_449785)
Kl_Yap3     170 NQTSDSATGAKDSEEQIRRKRALNRAAQKAFRERKEAR 207  (CAH02534)
Ca_Fcr3     197 AALIARDDSELTEEELQMKRKAQNRAAQRAFRERKESK 234  (AOW29611)
An_RsmA     104 SSSEEKDKDGIGITPAQSKRKAQNRAAQRAFRERKERH 141  (XP_662166)
Cn_Yap3     236 HEPRPAPPPEPPISNEKLSRAEQNKRAQQAFRRRREEH 273  (AFR92193)

Sc_Yap5      45 VHRLHEDYETEENDEELQKKKRQNRDAQRAYRERKNNK  82  (NP_012283)
Sk_Yap5/7   109 SGDEGNASGDENGVDSVEKRRKQNRDAQRAYRERRTTR 146  (EJT43076)
Cg_Yap5      64 PAGDSPTGEDSKHMSGPNRKRLQNREAQRAYRERSRNK 101  (XP_448602)
Sc_Yap7     112 SDDEGNGSGDENGVDSVEKRRRQNRDAQRAYRERRTTR 149  (NP_014614)
Cg_Yap7     183 DGDVEKNEYTLDMSDTAARRRRQNREAQRAYRERKATR 220  (XP_446029)
Kl_Yap5/7   126 SMDDSNQYFTSRSSNYAEKRRKQNREAQRAYRERKANK 163  (CAH00798)
Ca_Hap43     70 LPPRPKPGRKPSVDTPASKRKAQNRAAQRAFRERRATR 107  (AOW26746)
An_HapX      45 IPPRPKPGRKPATDTPPTKRKAQNRAAQRAFRERRAAR  83  (XP_681520)
Cn_HapX     121 LPERAKPGRKVSNDEPDNKRQSQNRLSQRAHRARRTDY 158  (XP_012049410)
                _________________  ___________________
                Extended homology     Basic region
                     region
```

Figure S1. Alignment of basic regions and the N-terminal adjacent sequences of fungal AP-1 proteins from *Saccharomyces cerevisiae* (Sc), *S. kudriavzevii* (Sk), *Candida albicans* (Ca), *C. glabrata* (Cg), *Kluyveromyces lactis* (Kl), *Aspergilus nidulans* (An) and *Cryptococcus neoformans* (Cn). Conserved amino acid residues involved in direct binding to DNA bases as determined for the *S. pombe* Pap1 protein are indicated at the top of sequence alignment. Identical or similar amino acid residues are highlighted accordingly. NCBI accession numbers for each protein sequence are indicated in the brackets.

Supplementary Figure S2

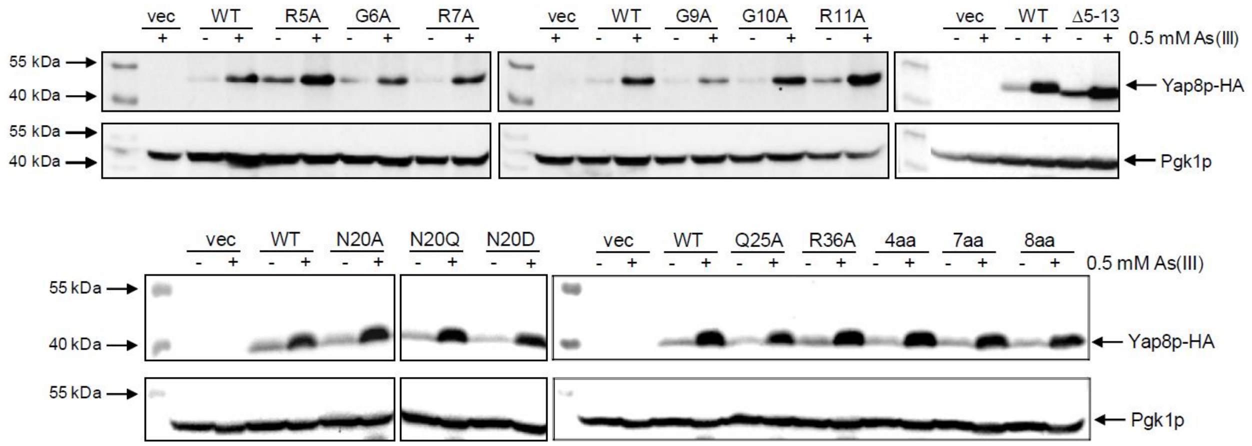

Figure S2. Protein level of Yap8 variants. Western blot analysis of total protein extracts prepared from the *yap8*Δ mutant transformed with the plasmids expressing indicated variants of Yap8-HA fusion protein under the control of constitutive *TPI1* promoter. Proteins were isolated from cells cultivated in standard conditions (control) or exposed to 0.5 mM As(III) for 30 min. The anti-HA antibodies were used to detect Yap8. Levels of 3-phosphoglycerate kinase Pgk1 detected with the anti-PGK1 antibodies served as a loading control.

Supplementary Figure S3

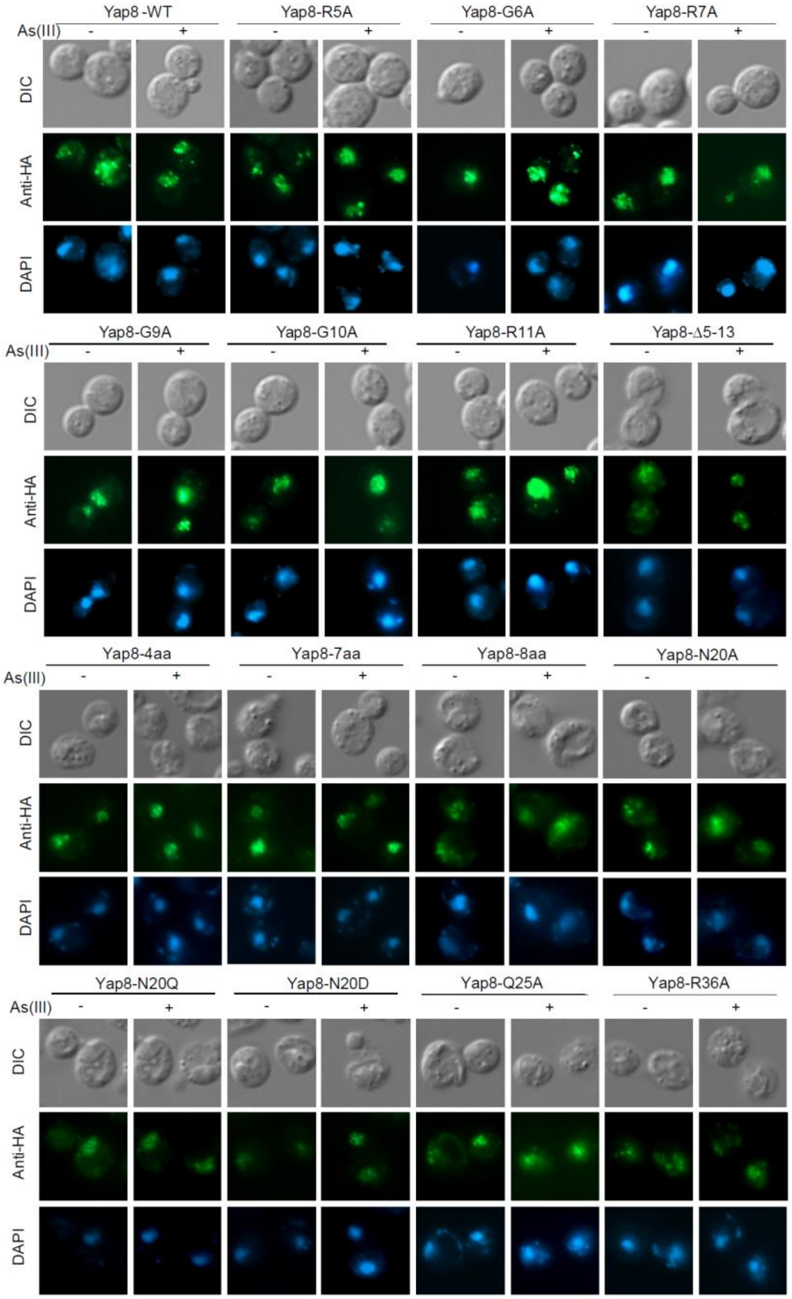

Figure S3. Subcellular localization of Yap8 variants. The *yap8*Δ mutant was transformed with pYX122-based plasmids expressing indicated mutant variants of Yap8-HA fusion protein under the control of constitutive *TPI1* promoter. Cells were cultivated in standard conditions (control) or exposed to 0.5 mM As(III) for 30 min and then subjected to immunofluorescence microscopy using primary anti-HA antibody and secondary Alexa Fluor® 488-labeled antibody to detect subcellular localization of mutant variants of Yap8-HA. Cells were also stained with DAPI to visualize nuclei and analyzed by differential interference contrast (DIC).

Supplementary Figure S4

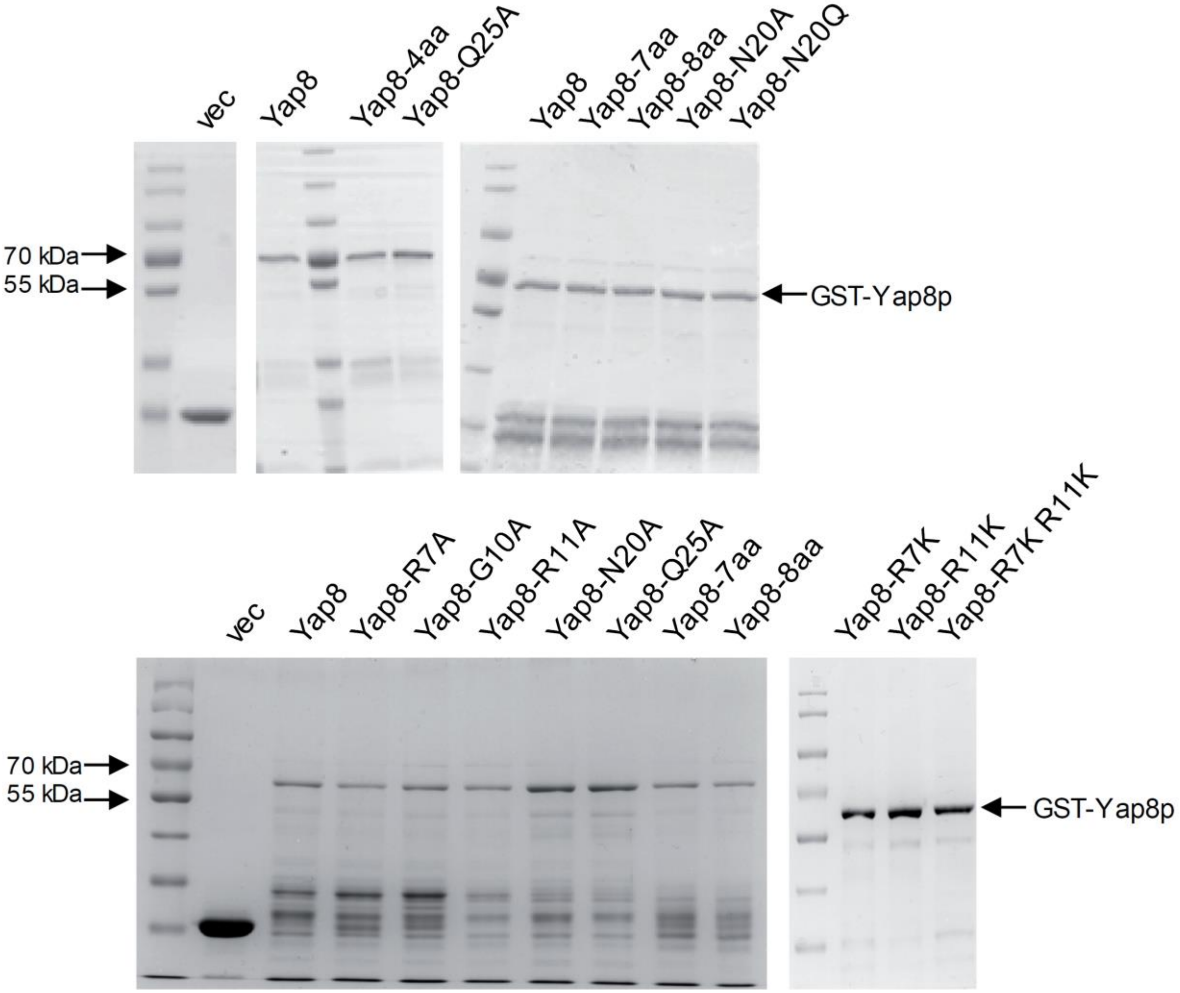

Figure S4. SDS-PAGE analysis of purified variants of GST-Yap8 proteins. Protein extracts of wild type and indicated mutant variants of GST-Yap8 fusion were isolated from *E. coli* and purified using glutathione sepharose affinity chromatography resin (GE Healthcare Life Sciences). Samples (~10 ng per lane) were loaded on a 10% polyacrylamide gel, subjected to SDS-PAGE and stained with Coomassie blue. PageRuler™ Prestained Protein Ladder (Thermo Scientific) was used as a protein marker. Vec sample is a GST protein alone. The upper panel refers to Figure 4 , the lower panel refers to Figure 3 and 5.

Supplementary Figure S5

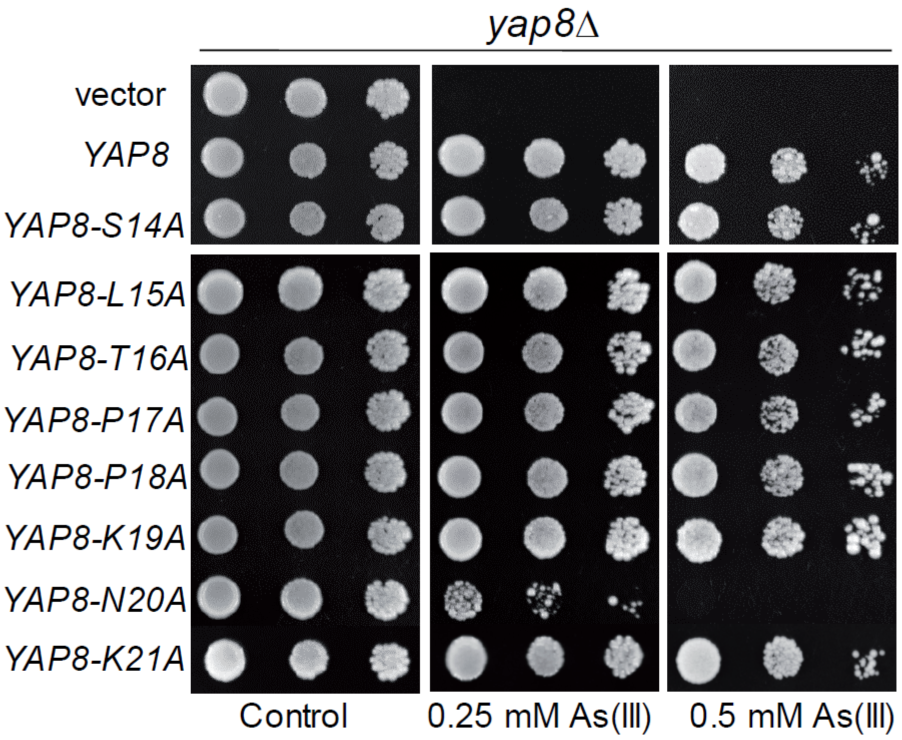

Figure S5. Mutational analysis of the N-terminal region adjacent to the basic region of Yap8. The *yap8*Δ mutant was transformed with empty vector (pYX122) or plasmids expressing indicated Yap8 variants. The resulting transformants were spotted on minimal selective plates containing various concentrations of As(III) and incubated 3 days at 28°C.

Supplementary Figure S6

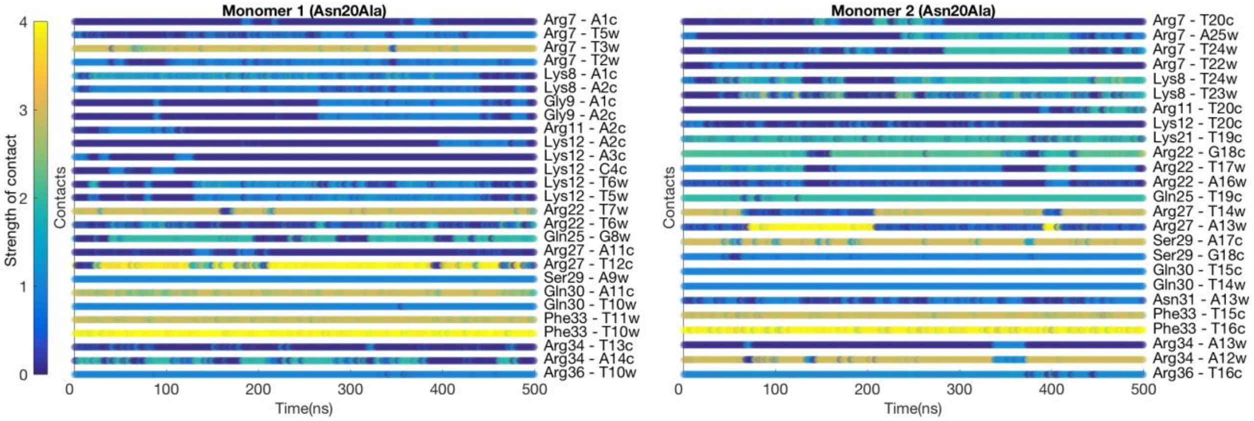

Figure S6. Dynamic interactions maps illustrating the intermolecular DNA-Yap8 (N20A) mutant interface. The interactions between pairs of the protein-DNA residues are characterized by a contact strength and its occurrence during the 0.5 μs MD simulation.
